# The ACORNS grading scale: a novel tool for the prediction of malignant brain edema after endovascular thrombectomy

**DOI:** 10.1136/jnis-2022-019404

**Published:** 2022-10-07

**Authors:** Xianjun Huang, Chu Chen, Huaiming Wang, Qiankun Cai, Zibao Li, Junfeng Xu, Lili Yuan, Xiangjun Xu, Qian Yang, Zhiming Zhou, Xinfeng Liu

**Affiliations:** 1 Department of Neurology, Yijishan Hospital of Wannan Medical College, Wuhu, Anhui Province, China; 2 Department of Neurology, The 80th Group Army Hospital of the People's Liberation Army, Weifang, Shandong Province, China; 3 Department of Neurology, Medical School of Nanjing University, Nanjing, Jiangsu Province, China; 4 Department of Neurology, The Second Affiliated Hospital of Fujian Medical University, Quanzhou, Fujian, China; 5 Stroke Center & Department of Neurology, University of Science and Technology of China, Hefei, Anhui Province, China

**Keywords:** Stroke, Thrombectomy

## Abstract

**Background:**

To develop and validate a novel tool for predicting the development of malignant brain edema (MBE) in large vessel occlusion stroke patients after endovascular thrombectomy (EVT).

**Methods:**

We used a prospectively registered population of EVT patients from three comprehensive stroke centers. The population was randomly divided into two subsets (7:3): a training cohort and an internal validation cohort. External validation was performed using the Endovascular Treatment for Acute Anterior Circulation Ischemic Stroke Registry in China (ACTUAL) database. MBE was defined as (1) hypodense parenchyma in at least 50% of the middle cerebral artery and signs of local brain swelling, and (2) a midline shift of ≥5 mm at the septum pellucidum or pineal gland with obliteration of the basal cisterns. The model was constructed using logistic regression analysis. The performance of the model was examined in terms of discrimination and calibration.

**Results:**

After adjusting for other confounders, baseline National Institutes of Health Stroke Scale (NIHSS) and Alberta Stroke Program Early CT (ASPECT) scores, a clinical history of hypertension, collateral status, intravenous thrombolysis before thrombectomy, fasting blood glucose, reperfusion status, and occlusion site were found to be independent predictors of MBE. These variables were combined to create the ACORNS grading scale. The areas under the curve in receiver operating curve analysis were 0.850 (95% CI 0.816 to 0.884), 0.874 (95% CI 0.821 to 0.926), and 0.785 (95% CI 0.740 to 0.829) for the training, internal validation, and external validation cohorts, respectively, indicating good discriminative performance in the validation cohorts.

**Conclusions:**

The ACORNS grading scale is an accurate and easily applicable model for the prediction of the development of MBE after EVT.

**WHAT IS ALREADY KNOWN ON THIS TOPIC**

- Malignant brain edema (MBE) is one of the important factors affecting the prognosis of endovascular thrombectomy (EVT) treatment. Close monitoring of patients with a high risk of MBE and early initiation of decompressive hemicraniectomy (DHC) warnings may further improve EVT patients’ functional outcomes. However, available data on predictors of MBE in EVT patients remain limited.

**WHAT THIS STUDY ADDS**

- We identified eight variables that could be obtained immediately at the end of the EVT procedure: clinical history of hypertension, intravenous thrombolysis, baseline National Institutes of Health Stroke Scale (NIHSS) and Alberta Stroke Program Early CT (ASPECT) scores, collateral circulation status, fasting blood glucose level, reperfusion status, and occlusion site. These variables were combined to develop the ACORNS grading scale. The ACORNS grading scale showed good discriminative performance and model fit in both validation cohorts.

**HOW THIS STUDY MIGHT AFFECT RESEARCH, PRACTICE OR POLICY**

- In clinical practice, the ability of the ACORNS grading scale to identify accurately EVT patients at high risk of MBE at their bedside is crucial for physicians and will facilitate the selection of appropriate treatment strategies and the close monitoring and triage of DHC.

## Introduction

Malignant brain edema (MBE) is one of the most serious complications in ischemic stroke patients; it can significantly affect prognosis and has a mortality rate of 40–80%.^1^ In recent years, endovascular thrombectomy (EVT) has been the standard of care for patients with acute anterior circulation large vessel occlusion stroke (LVOS).^2^ Nonetheless, a substantial number of patients treated with EVT do not gain functional independence. Previous studies have indicated that MBE is one of the important factors affecting the prognosis of EVT treatment.^3^ Although rapid and successful vascular recanalization through EVT can effectively reduce the incidence of MBE,^4^ MBE is still not uncommon in patients treated with EVT.^3^ Previous randomized controlled studies have confirmed that decompressive hemicraniectomy (DHC) within 48 hours of symptom onset is an effective treatment to reduce morbidity and mortality in patients with MBE.^5^ Therefore, close monitoring of patients with a high risk of MBE and early initiation of DHC warnings may further improve EVT patients’ functional outcomes.

Several studies have tried to develop prediction models for MBE in ischemic stroke, including the Kasner score^6^ (history of hypertension, history of heart failure, elevated white blood cell count, middle cerebral artery (MCA) hypodensity, and involvement of additional vascular territories) and EDEMA (Enhanced Detection of Edema in Malignant Anterior circulation stroke) score^7^ (basal cistern effacement, glucose level, intervention with tissue-type plasminogen activator or thrombectomy, midline shift, and history of previous stroke). However, these two models were based on patients who have not undergone a thrombectomy. Although the MBE score^8^ (baseline National Institutes of Health Stroke Scale (NIHSS) score, Alberta Stroke Program Early CT (ASPECT) score, collateral circulation, and revascularization) was developed in the EVT population, it was based on relatively small sample sizes and has not been externally validated. Therefore, available data on predictors of MBE in EVT patients remain limited.

In this study, we aimed to develop and validate a novel model to predict the occurrence of MBE in EVT patients immediately after the procedure in a bedside setting.

## Methods

### Study population

We enrolled patients with anterior circulation LVOS who underwent EVT at three comprehensive stroke centers (Jinling Hospital, Yijishan Hospital, and the Second Affiliated Hospital of Fujian Medical University) between January 2014 and July 2021.

Patients were enrolled if they fulfilled the following inclusion criteria: (1) treatment with EVT; (2) age ≥18 years; and (3) occlusion of the internal carotid artery (ICA) or of the proximal segment (M1) of the MCA confirmed by preoperative imaging. We excluded patients with occlusion of the distal segment of the MCA, anterior cerebral artery (ACA) occlusion, multiple vessel occlusion, and those without imaging data. However, patients with tandem occlusion (ICA and M1) were included in the study.

### External validation cohort

The ACTUAL (Endovascular Treatment for Acute Anterior Circulation Ischemic Stroke Registry in China) database was used as the external validation cohort for the predictive model. The ACTUAL registry is a multicenter registry program for patients treated with EVT from January 2014 to June 2016 and involves 21 stroke centers in 10 provinces across China.^9^ The institutional review board of each participating center approved the research protocol, and informed consent was waived because of the retrospective nature of this study. Details of the ACTUAL database have been previously reported.^9^ The inclusion criteria for the ACTUAL registry were as follows: (1) age ≥18 years; (2) treatment with EVT; and (3) anterior circulation LVOS confirmed by preoperative imaging. We excluded patients with occlusion of the ACA and of the distal segment of the MCA. Additionally, patients with incomplete baseline critical data (eg, ASPECT score and MBE data) were excluded. A total of 95 patients overlapped between the ACTUAL database and the derivation cohort. In the external validation cohort, we also excluded the 95 patients due to overlapping with the derivation cohort.

### Data collection

Baseline clinical data, including demographics, medical history, baseline NIHSS and ASPECT scores, the Trial of ORG 10172 in Acute Stroke Treatment (TOAST) classification, time from stroke onset to puncture (OTP), and time from stroke onset to reperfusion (OTR), were collected for analysis.

The procedural parameters, including occlusion site, EVT approach, bridging treatment, recanalization status, and collateral circulation, were evaluated by two experienced operators. In the event of discrepancies, the final result was determined by consensus opinion. Recanalization status was evaluated based on the modified Thrombolysis In Cerebral Infarction (mTICI) grading system. Successful recanalization was defined as an mTICI score of 2b or 3. Good collaterals were defined as >50% filling of the occluded area based on the preoperative angiography.^10^

### Definition of MBE

Following a previous study,^4^ MBE after EVT was defined according to the following criteria: (1) at least 50% of the MCA had hypodense parenchyma and signs of local brain swelling, such as disappearance of the sulcus and compression of the lateral ventricle; and (2) a midline shift of ≥5 mm at the septum pellucidum or pineal gland with obliteration of the basal cisterns was present. MBE was assessed on follow-up imaging 3–5 days after EVT. For all enrolled subjects, the imaging characteristics were evaluated by two neurologists/interventionalists who were blinded to the clinical information.

### Statistical analysis

A subset of approximately 70% of the patients were randomly selected to create a training cohort, and the remaining patients constituted the internal validation cohort. We compared baseline clinical and imaging characteristics between patients with and without MBE in the training cohort. Continuous variables are expressed as mean±SD or as median (IQR). Categorical variables are expressed as percentages. Continuous variables were analyzed using the Mann-Whitney U test. Categorical variables were analyzed using the χ^2^ test or Fisher’s exact test as appropriate. For the missing variables, multiple imputations (10 datasets) with multivariate regression analysis were used. The missing data of the cohorts are shown in online supplemental table I.

10.1136/jnis-2022-019404.supp1 Supplementary data

To identify the independent predictors of the development of MBE, we implemented a logistic regression analysis including the variables with p<0.05 in the univariate analysis of the training cohort. Regression coefficients and odds ratios (ORs) with two-sided 95% confidence intervals (95% CIs) for each of the variables included in the model were then calculated. According to the regression coefficients of individual covariables in the model, we calculated the corresponding points in the scale and derived a scoring system.

The performance of the scoring system was assessed in terms of discrimination and calibration. Discrimination is the ability to distinguish between patients with MBE versus those without MBE and was examined using the area under the receiver operating characteristic (ROC) curve with the corresponding 95% CI. A clinically useful area under the curve (AUC) was considered to be approximately ≥0.70. Calibration was assessed with calibration plots and with the Hosmer-Lemeshow test.

We further compared the new scoring system to a previously published risk model (the MBE score).^11^ All statistical analyses were computed using STATA version 15 (StataCorp, College Station, TX) and SPSS 25 (IBM Corp, Armonk, NY).

## Results

**Figure 1:**
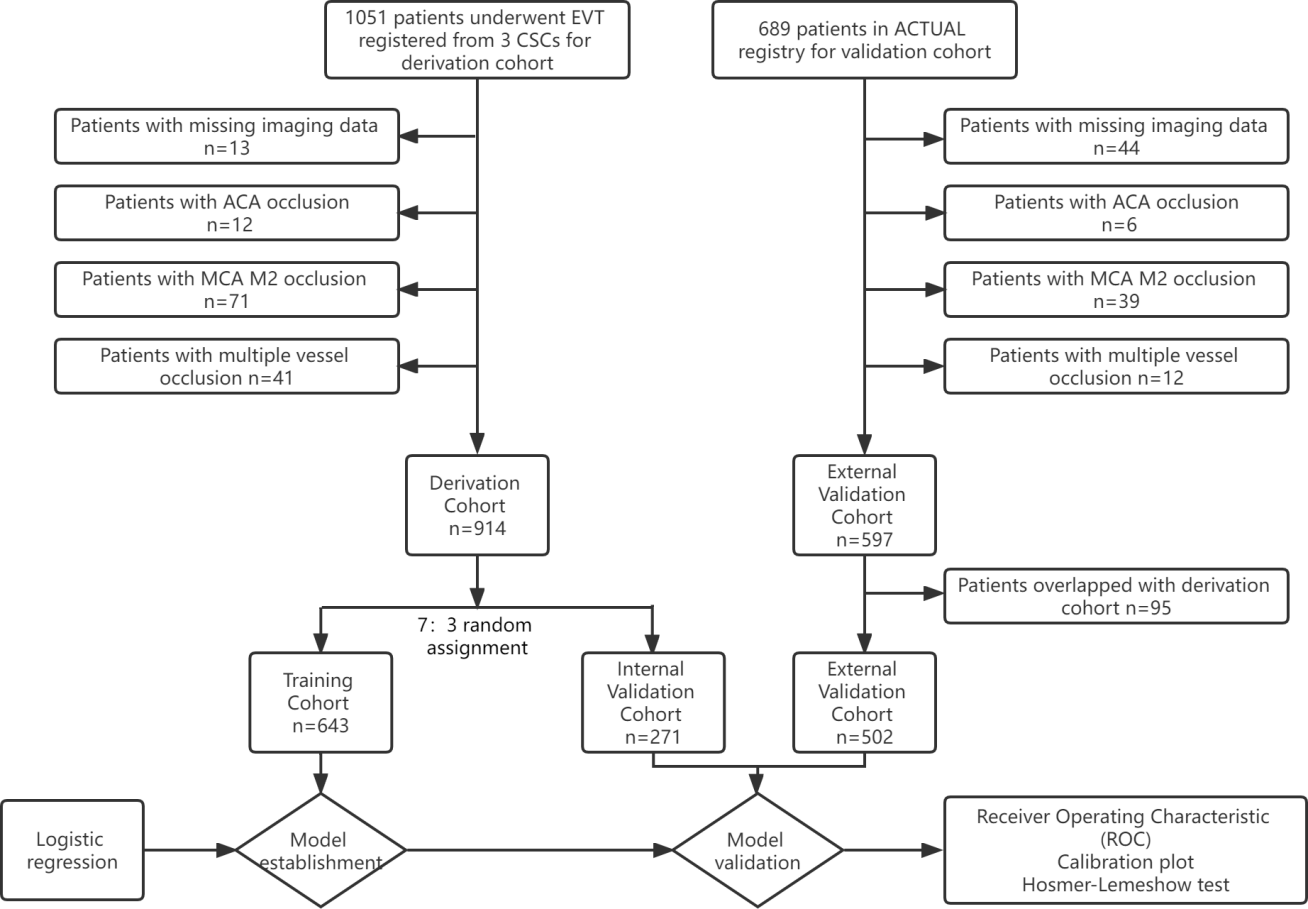
Flow chart of the inclusion data for the study population. ACA, anterior cerebral artery; ACTUAL, Endovascular Treatment for Acute Anterior Circulation Ischemic Stroke Registry in China; ASPECT, Alberta Stroke Program Early CT; CSCs, Comprehensive Stroke Centers; EVT, endovascular thrombectomy; MCA, middle cerebral artery.

Of the 1051 patients from three comprehensive stroke centers, 914 patients met the inclusion criteria and were randomly assigned to either the training set (n=643) or the internal validation set (n=271). The flow chart for inclusion in this study is presented in figure 1. The baseline characteristics and procedural parameters for the study population are shown in table 1.

### Patient characteristics

In the training cohort: the mean age of the patients was 66.6±11.9 years; 61.3% (n=394) were men; 23.2% (n=149) received bridging treatment; and 21.0% (n=135) developed MBE. The mean age of the patients was 66.4±11.3 years in the internal validation cohort and 65.1±12.6 years in the external validation cohort. MBE was observed in 21.0% and 25.9% of the patients in the internal and external validation cohorts, respectively. Additionally, symptomatic intracranial hemorrhage (sICH) was observed in 9.0% (n=58) and 14.4% (n=39) of the patients in the training and internal validation cohorts (p=0.016), respectively. A comparison of baseline characteristics among the three cohorts is shown in table 1.

**Table 1:** Baseline characteristics of the cohorts

	Training cohort	Internal validation cohort	P value*	External validation cohort	P value†
	n=643	n=271		n=502	
Age, years, mean (SD)	66.6 (11.9)	66.4 (11.3)	0.807	65.1 (12.6)	0.035
Men, n (%)	394 (61.3)	172 (63.5)	0.533	305 (60.8)	0.858
Hypertension, n (%)	428 (66.6)	171 (63.1)	0.314	312 (62.2)	0.121
Diabetes, n (%)	111 (17.3)	51 (18.8)	0.574	95 (18.9)	0.468
Atrial fibrillation, n (%)	297 (46.2)	137 (50.6)	0.228	207 (41.2)	0.1
Baseline NIHSS score, median (IQR)	16 (13–20)	15 (12–19)	0.231	16 (12–21)	0.286
Baseline ASPECT score, median (IQR)	9 (8–10)	9 (7–10)	0.769	9 (8–10)	0.001
IVT, n (%)	149 (23.2)	54 (19.9)	0.281	164 (32.7)	<0.001
TOAST classification, n (%)			0.262		<0.001
Atherosclerotic	222 (34.5)	86 (31.7)		224 (44.6)	
Cardioembolic	337 (52.4)	157 (57.9)		248 (49.4)	
Others	84 (13.1)	28 (10.3)		30 (6.0)	
OTP, min, median (IQR)	279 (218–350)	292 (215–360)	0.586	280 (205–360)	0.535
OTR, min, median (IQR)	359 (289–446)	368 (300–455)	0.241	395 (307–495)	0.001
Collateral status, n (%)			0.256		0.254
Poor	351 (54.6)	159 (58.7)		257 (51.2)	
Good	292 (45.4)	112 (41.3)		245 (48.8)	
First treatment, n (%)			0.930		<0.001
Stent retriever	427 (66.4)	177 (65.3)		469 (93.4)	
Contact aspiration	133 (20.7)	59 (21.8)		0	
Angioplasty	82 (12.8)	35 (12.9)		33 (6.6)	
Tandem, n (%)	69 (10.7)	36 (13.3)	0.269	44 (8.8)	0.069
Occlusion site, n (%)			0.471		0.541
MCA	356 (55.4)	143 (52.8)		215 (42.8)	
ICA	287 (44.6)	128 (47.2)		287 (57.2)	
mTICI 2b-3, n (%)	532 (82.7)	203 (74.9)	0.006	427 (85.1)	0.290
MBE, n (%)	135 (21.0)	57 (21.0)	0.990	130 (25.9)	0.051
FBG, mmol/L, mean (SD)	7.17 (3.18)	7.46 (7.10)	0.411	7.82 (3.15)	0.001
LDL, mmol/L, mean (SD)	2.36 (0.84)	2.30 (0.89)	0.307	2.57 (0.86)	<0.001
BUN, mmol/L, mean (SD)	6.22 (2.83)	7.70 (26.6)	0.217	6.25 (4.29)	0.232
Cr, μmol/L, mean (SD)	83.3 (40.40)	80.38 (37.93)	0.268	80.47 (38.26)	0.264
sICH, n (%)	58 (9)	39 (14.4)	0.016		

*P indicates training cohort compared with internal validation cohort.

†P indicates training cohort compared with external validation cohort.

ASPECT, Alberta Stroke Program Early CT; BUN, blood urea nitrogen; Cr, creatinine; FBG, fasting blood glucose; ICA, internal carotid artery; IVT, intravenous thrombolysis; LDL, low-density lipoprotein; MBE, malignant brain edema; MCA, middle cerebral artery; mTICI, modified Thrombolysis In Cerebral Infarction; NIHSS, National Institutes of Health Stroke Scale; OTP, symptom onset to groin puncture time; OTR, time from stroke onset to recanalization; sICH, symptomatic intracranial hemorrhage; TOAST, the Trial of ORG 10172 in Acute Stroke Treatment.

### Model development

In the training cohort, compared with the non-MBE group, the MBE group had a significantly higher baseline NIHSS score (p<0.001), higher fasting blood glucose (FBG) level (p<0.001), and lower baseline ASPECT score (p<0.001). Moreover, the rates of history of hypertension (p=0.001), ICA occlusion (p<0.001), poor collateral circulation (p<0.001), and incomplete recanalization (p<0.001) were significantly higher in the MBE group. Additionally, patients who received intravenous alteplase plus EVT developed MBE more frequently than patients who received EVT alone (p=0.014). The results of the univariable and multivariable logistic regression for MBE after EVT in the training cohort are shown in table 2.

**Table 2:** Results of the univariable and multivariable logistic regression for MBE after endovascular thrombectomy in the training cohort

	Univariable			Multivariable		
	MBE	Non-MBE	P value	Regression coefficient	OR (95% CI)	P value
	n=135	n=508				
Age, years, mean (SD)	66 (12.1)	67 (11.4)	0.427			
Men, n (%)	81 (60.0)	313 (61.6)	0.732			
Hypertension, n (%)	106 (78.5)	322 (63.4)	0.001	0.704	2.022 (1.184 to 3.451)	0.010
Diabetes, n (%)	29 (21.5)	82 (16.1)	0.145			
Atrial fibrillation, n (%)	69 (51.1)	228 (44.9)	0.197			
Baseline SBP, mm Hg, median (IQR)	150 (133–165)	149 (129–160)	0.256			
Baseline DBP, mm Hg, median (IQR)	84 (72–92)	81 (74–91)	0.714			
Baseline NIHSS score, median (IQR)	18 (16–22)	15 (12–19)	<0.001	0.052	1.053 (1.010 to 1.099)	0.015
Baseline ASPECT score, median (IQR)	8 (5–9)	9 (8–10)	<0.001	−0.271	0.763 (0.682 to 0.852)	<0.001
IVT, n (%)	42 (31.1)	107 (21.1)	0.014	0.602	1.825 (1.095 to 3.042)	0.021
TOAST classification, n (%)			0.090			
Atherosclerotic	36 (26.7)	186 (36.6)				
Cardioembolic	78 (57.8)	259 (51.0)				
Others	21 (15.6)	63 (12.4)				
OTP, min, median (IQR)	279 (218–330)	279 (219–359)	0.697			
OTR, min, median (IQR)	380 (310–452)	355 (285–445)	0.127			
Collateral status, n (%)			<0.001			
Poor	111 (82.2)	240 (47.5)		−1.106	Reference	
Good	24 (17.8)	268 (52.8)			0.331 (0.195 to 0.561)	<0.001
First-line treatment, n (%)			0.078			
Stent retriever	89 (65.9)	338 (66.7)				
Contact aspiration	35 (25.9)	98 (19.3)				
Angioplasty or stent	11 (8.1)	71(14)				
mTICI 2b-3, n (%)	87 (64.4)	445 (87.6)	<0.001	−1.095	0.334 (0.199 to 0.563)	<0.001
Tandem, n (%)	21 (15.6)	85 (16.7)	0.743			
Occlusion site, n (%)			<0.001			
MCA	42 (31.1)	314 (61.8)		−0.962	0.382 (0.241 to 0.606)	<0.001
ICA	93 (68.9)	194 (38.2)			Reference	
FBG, mmol/L, median (IQR)	7.7 (6.0–10.1)	6.0 (5.1–7.6)	<0.001	0.117	1.124 (1.051 to 1.202)	0.001
LDL, mmol/L, median (IQR)	2.3 (1.8–2.9)	2.3 (1.9–2.9)	0.691			
BUN, mmol/L, median (IQR)	5.7 (4.6–8.0)	5.6 (4.5–7.1)	0.321			
Cr, μmol/l, median (IQR)	75.0 (63.0–99.5)	74.0 (60.7–92.9)	0.187			

ASPECT, Alberta Stroke Program Early CT; BUN, blood urea nitrogen; Cr, creatinine; DBP, diastolic blood pressure; FBG, fasting blood glucose; ICA, internal carotid artery; IVT, intravenous thrombolysis; LDL, low-density lipoprotein; MBE, malignant brain edema; MCA, middle cerebral artery; mTICI, modified Thrombolysis In Cerebral Infarction; NIHSS, National Institutes of Health Stroke Scale; OTP, symptom onset to groin puncture time; OTR, time from stroke onset to recanalization; SBP, systolic blood pressure; TOAST, the Trial of ORG 10172 in Acute Stroke.

**Figure 2:**
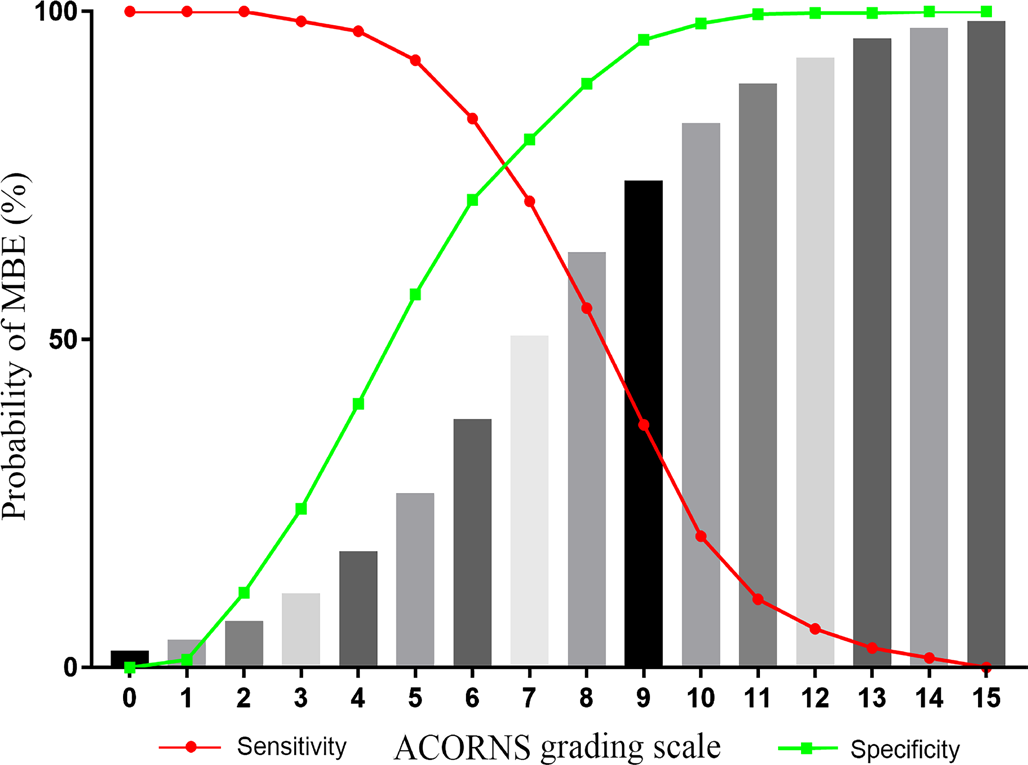
Probability of developing malignant brain edema (MBE) as predicted by the model (gray bars).

The logistic regression analysis showed that a history of hypertension, baseline ASPECT scores, baseline NIHSS scores, IVT, collateral circulation, baseline FBG level, reperfusion status, and occlusion site were independent predictors for the occurrence of MBE (table 1). The p value of the Hosmer-Lemeshow test was 0.530. The AUC was 0.845 (95% CI 0.812 to 0.878, p<0.001). Based on the β coefficients obtained in table 2, the following model was derived: y=1+0.704 (history of hypertension) +0.602 (IVT) – 0.271 (ASPECT score) – 1.106 (collateral status) + 0.117 (FBG) – 1.095 (reperfusion status) + 0.052 (NIHSS score) – 0.962 (occlusion site). Then, according to the model, we derived the ACORNS score to predict the risk of MBE (table 3). The final score was derived by summing the points of each predictor, with a maximum achievable score of 15. The risk of presenting with MBE is given by $P_{MBE}$ = $\frac{e^{Y}}{1+e^{Y}}$ . A patient with a score of 0 (no risk factors) has $P_{MBE}=$ 2.6%, and a patient with a full score of 15 has $P_{MBE}=$ 98.5%. A stepwise progression of increased MBE probability was noted as the score value increased (figure 2).

**Table 3:** Components of the ACORNS grading scale

Item	Categories	Points
Baseline **A**SPECT score	8–10	0
	6–7	1
	<6	3
**C**ollateral circulation	Good	0
	Poor	2
Fast bl**O**od glucose	≤7.0	0
	7.1–11.1	1
	>11.1	2
History of hypertension (blo**O**d pressure)	No	0
	Yes	1
IVT before thrombectomy (**r**t-PA)	No	0
	Yes	1
**R**eperfusion status (mTICI)	2b-3	0
	0–2a	2
Baseline **N**IHSS score	<13	0
	13–20	1
	>20	2
Occlusion **S**ite	MCA	0
	ICA	2

ASPECT, Alberta Stroke Program Early CT; ICA, internal carotid artery; IVT, intravenous thrombolysis; MCA, middle cerebral artery; mTICI, modified Thrombolysis In Cerebral Infarction; NIHSS, National Institutes of Health Stroke Scale; rt-PA, recombinant tissue plasminogen activator.

**Figure 3:**
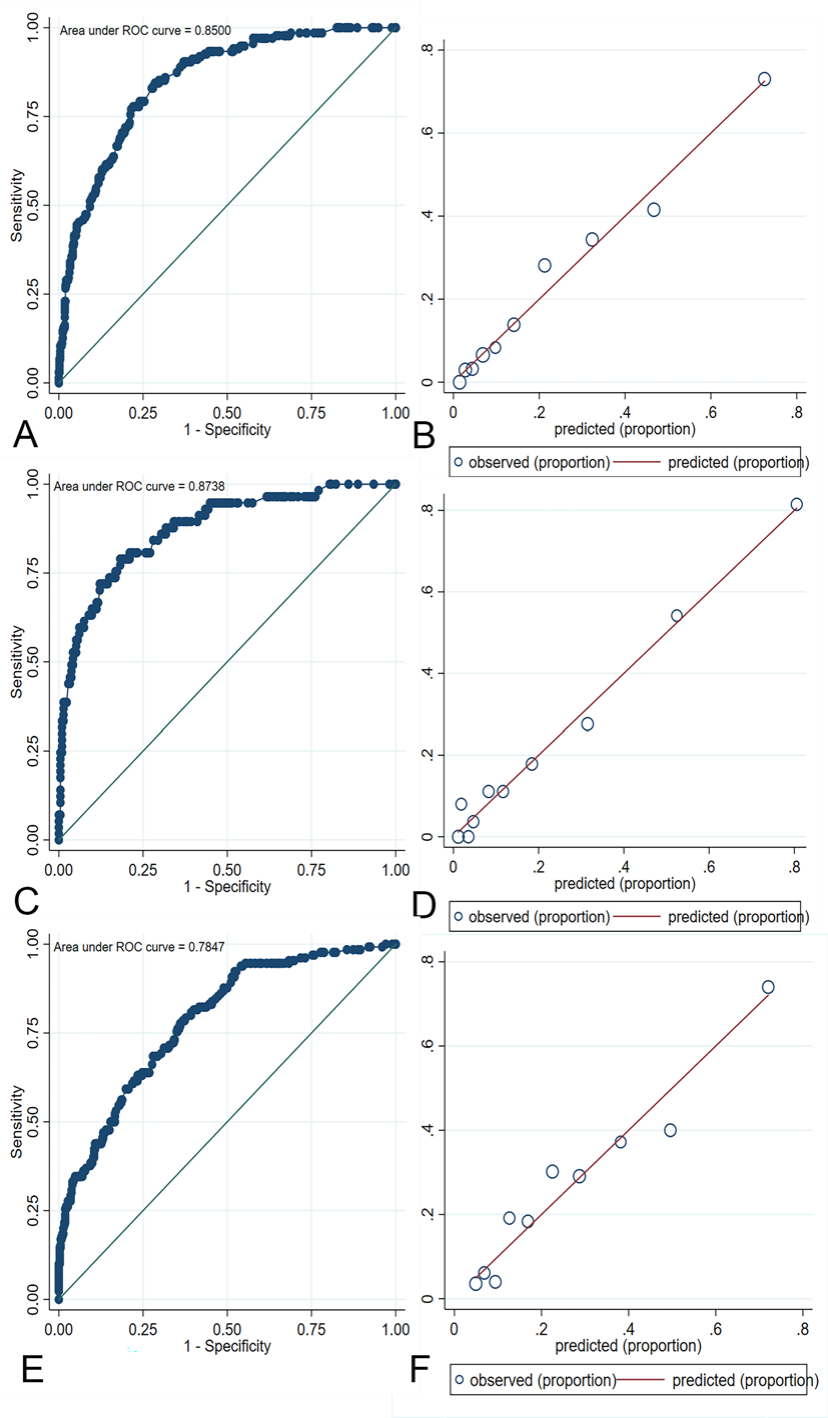
Evaluation of model discrimination and calibration in the training cohort (A, B), internal validation cohort (C, D) and external validation cohort (E, F). ROC, receiver operating characteristic.

Based on the ACORNS score to predict the risk of MBE, an AUC of 0.850 (95% CI 0.816 to 0.884, p<0.001) was obtained when testing the training dataset (figure 3A). The p value of the Hosmer-Lemeshow test was 0.812 (figure 3B), indicating appropriate goodness-of-fit. An optimal cut-off of 7 (optimal criterion with 71.1% sensitivity and 80.5% specificity) revealed the ACORNS grading scale to have good predictive accuracy. The MBE rates according to the ACORNS score are shown in online supplemental table III and figure I.

### Model validation

To assess the performance of the ACORNS score, two validation cohorts were used to verify the model. In the internal validation cohort, the model showed an AUC of 0.874 (95% CI 0.821 to 0.926, p<0.001), and the p value of the Hosmer-Lemeshow test was 0.477 (figure 3C,D).

In the external validation cohort (ACTUAL database), ROC analysis revealed a reliable prediction of MBE with an AUC of 0.785 (95% CI 0.740 to 0.829, p<0.001), thus indicating good predictive accuracy of the new risk grading scale (figure 3E). The Hosmer-Lemeshow test yielded a p value of 0.385, and figure 3F shows the calibration graph exhibiting the model fit.

### Comparison with the MBE score

**Figure 4:**
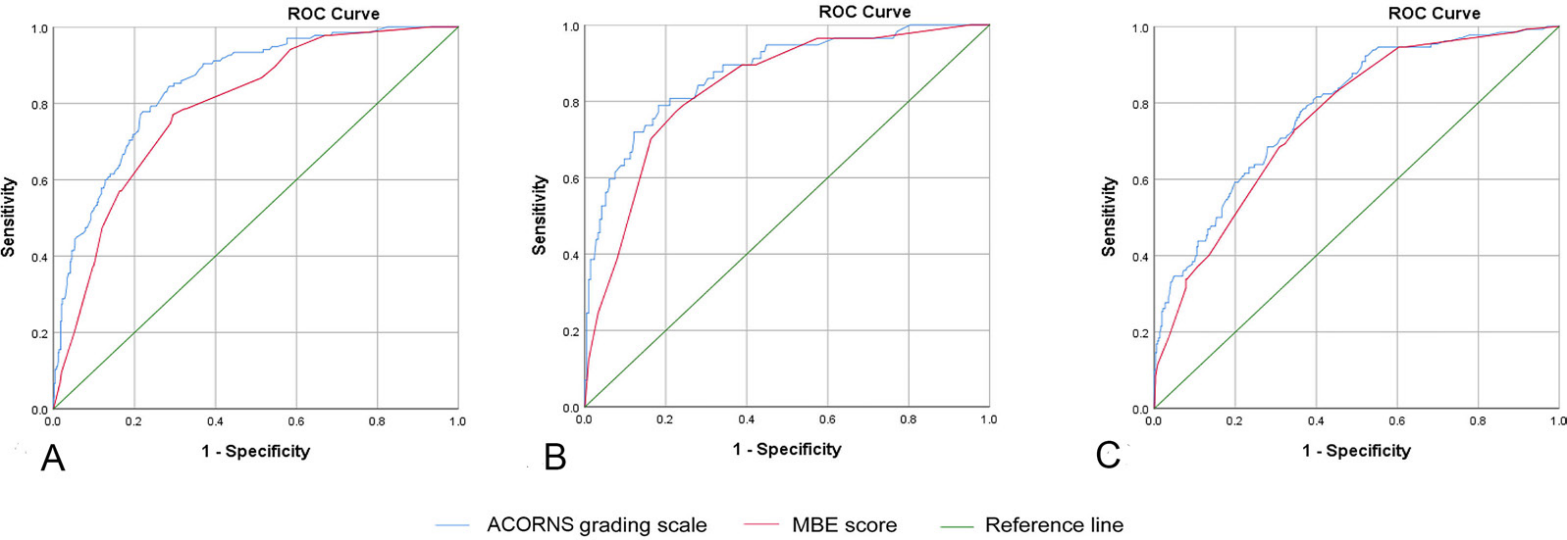
Comparison of the discriminative performance of the ACORNS grading scale with the MBE score. (A) Comparison with the training cohort. (B) Comparison with the internal validation cohort. (C) Comparison with the external validation cohort. MBE, malignant brain edema; ROC, receiver operating characteristic.

To illustrate the discriminative performance of the ACORNS score, we further compared the model with the previous MBE score. The AUC of the ACORNS grading scale was superior to that of the MBE score in the training cohort (0.850, 95% CI 0.816 to 0.884 vs 0.790, 95% CI 0.750 to 0.830, p<0.001) (figure 4A) and in the internal validation cohort (0.874, 95% CI 0.821 to 0.926 vs 0.836, 95% CI 0.779 to 0.892, p=0.035) (figure 4B). In the external validation cohort, the AUC of the ACORNS grading scale was better than that of the MBE score; unfortunately, there was no significant statistical difference (0.785, 95% CI 0.740 to 0.829 vs 0.760, 95% CI 0.714 to 0.805, p=0.131) (figure 4C).

## Discussion

We aimed to develop a model to predict the occurrence of MBE in patients with EVT. To be considered clinically useful, the prediction model needs to include parameters that are available easily and early. We identified eight variables that could be obtained immediately at the end of the EVT procedure: clinical history of hypertension, IVT, baseline ASPECT and NIHSS scores, collateral circulation status, FBG level, reperfusion status, and occlusion site. These variables were combined to develop the ACORNS grading scale. The ACORNS grading scale showed good discriminative performance and model fit in both validation cohorts.

### Mechanisms of associations

The association between baseline NIHSS and ASPECT scores and the development of MBE is not unexpected and has been previously confirmed,^8^ as higher scores often indicate massive cerebral infarction. A recent meta-analysis also showed that high NIHSS scores and large parenchymal hypoattenuation on CT are reliable early predictors of MBE.^12^

Collateral circulation plays an important role in the pathophysiology of cerebral ischemia. Poor collateral circulation is significantly associated with malignant MCA evolution after reperfusion therapy and is related to the rate of early edema progression in acute ischemic stroke.^11^ It is possible that poor collateral circulation accelerates the recruitment of the ischemic penumbra into infarction, leading to a larger infarct size.

The association between a clinical history of hypertension and MBE has been reported in a prior study.^13^ Furthermore, higher mean systolic blood pressure during the first 24 hours after a stroke or an EVT procedure is associated with the development of MBE.^14^ We speculated that hypertension may facilitate edema formation by chronic impairments in cerebral collateral flow and autoregulation,^15^ increasing blood‒brain barrier permeability^16^ and promoting a deleterious proinflammatory state.^17^

An elevated blood glucose level at admission is related to poor clinical outcomes in EVT patients.^18^ Hyperglycemia may have deleterious effects on vascular integrity through a variety of mechanisms, including blood‒brain barrier damage, poor collateral circulation, excitatory chemokines, and acidosis, all of which would result in a higher risk of MBE.^19^ Broocks *et al* observed an interrelation among elevated blood glucose, poor collateral circulation, and pronounced early ischemic edema.^20^ The potential mechanism was that hyperglycemia could probably reduce the recruitment of new collateral flow after acute arterial occlusion.^10 20^ In our study, we chose FBG as the predictor, mainly because FBG is less affected by factors than admission blood glucose level.

The relationship between the location of vessel occlusion and the development of MBE has rarely been explored. We previously identified ICA occlusion as an independent risk factor for the development of MBE, even in patients with successful reperfusion.^3^ Furthermore, Thomalla *et al* obtained similar results in a prospective multicenter study.^21^ This finding could be explained by the fact that ICA occlusion not only reduces collateral compensation but also indicates a large burden of thrombus, which reduces the successful recanalization rate and prolongs the procedure time. The association between reperfusion status and MBE is noteworthy. Early studies suggested that revascularization may cause secondary reperfusion brain edema.^22 23^ However, several recent studies have demonstrated that recanalization can reduce the incidence of brain edema.^4 24^

One other major predictor of MBE in our study was IVT before thrombectomy. The association between IVT and MBE due to stroke is unclear and has not been described in previous studies. Two mechanisms can explain this phenomenon. First, IVT before thrombectomy could affect the workflow of stroke treatment to a certain extent and prolong the time to achieve successful reperfusion.^25 26^ Second, the increased risk of hemorrhagic transformation after thrombolysis may increase the incidence of MBE.^27^ We performed a post-hoc sensitivity analysis and showed that when the patients who developed sICH in the derivation cohort were excluded, IVT before thrombectomy was no longer a predictor of MBE (online supplemental table II). Our findings provide fresh insights into a topic that has yielded conflicting results between real world research and randomized controlled study.

### Clinical implications

The ACORNS grading scale showed good discriminative performance and model fit in its validation with a nationwide database. In addition, when compared with the MBE scale, the current model provides greatly improved discrimination.

In clinical practice, the ability of the ACORNS grading scale to identify accurately EVT patients at high risk of MBE at their bedside is crucial for physicians and will facilitate the selection of appropriate treatment strategies and the close monitoring and triage of DHC.

The ACORNS grading scale suggests the factors, including core infarct volume (ASPECTS), blood glucose and reperfusion status, can be targets for intervention in patients at high risk of MBE. The rapid identification of stroke, acceleration of the reperfusion treatment process, and shortening of the recanalization time can reduce the growth of the infarct core. Improving the rate of recanalization may further reduce the occurrence of MBE. Additionally, for the group at high risk for MBE, strengthening perioperative management may promote early recognition of MBE. Moreover, perioperative blood pressure management of the group at high risk for MBE may be different from that of the general population. Therefore, the ACORNS grading scale provides convenience for the risk stratification of EVT patients.

### Strengths and limitations

It is worth noting that, in the external validation cohort, our model did not exhibit considerably better discrimination compared with the MBE score model. Moreover, the ACORNS score seems to be relatively more complex than the MBE score. The main reason was that the cohort used for the development of the ACORNS score was a relatively large unselected multicenter cohort of EVT patients. We did not exclude patients with hemorrhagic transformation, which may affect the evaluation of MBE and increase the incidence of MBE. In other words, the strength of the ACORNS score is more applicable to the real world. Another strength of the ACORNS score is that the model showed superior performance in both the internal and external validation for patients with EVT, which was lacking in published predictive models. Moreover, all of the identified predictors can be obtained early and easily, which makes this score a useful bedside predictive tool.

This study also has some limitations. First, the amount of missing data might cause bias in the development and validation of the model. Second, we did not perform a comparison with other scores, such as the EDEMA score and the modified EDEMA score.^7 8^ An important reason for this is the heterogeneity of the enrolled patients and the different predictive factors used in different studies. The elements of the EDEMA score and the modified EDEMA score, including the midline shift and basal cistern effacement, are relative contraindications for the EVT procedure. Additionally, previous studies indicated that hyperattenuating lesions after thrombectomy was an important imaging marker for sICH and the final infarction size for patients with EVT.^28 29^ We speculated that the volume of brain parenchyma with hyperattenuation after EVT may also predict the development of MBE. However, we did not include this imaging marker as a predictor. The main reasons were as follows: on the one hand, the formation mechanism of postoperative hyperattenuating lesions is not clear; on the other hand, there is a lack of uniformity in the evaluation of hyperattenuating lesions after thrombectomy.

### Conclusion

The ACORNS grading scale, which consists of a clinical history of hypertension, baseline NIHSS and ASPECT scores, glycemia, collateral circulation, occlusion location, IVT before thrombectomy, and reperfusion status, is an accurate, generalizable, and easily applicable model for the early prediction of MBE after EVT. Further studies are warranted to validate the effectiveness of this model in other populations.

10.1136/jnis-2022-019404.supp2 Supplementary data

## Data Availability

Data are available upon reasonable request.

## References

[1] Balami JS , Chen R-L , Grunwald IQ , et al . Neurological complications of acute ischaemic stroke. Lancet Neurol 2011;10:357–71. 10.1016/S1474-4422(10)70313-6 21247806

[2] Campbell BCV , Donnan GA , Lees KR , et al . Endovascular stent thrombectomy: the new standard of care for large vessel ischaemic stroke. Lancet Neurol 2015;14:846–54. 10.1016/S1474-4422(15)00140-4 26119323

[3] Huang X , Yang Q , Shi X , et al . Predictors of malignant brain edema after mechanical thrombectomy for acute ischemic stroke. J Neurointerv Surg 2019;11:994–8. 10.1136/neurintsurg-2018-014650 30798266

[4] Kimberly WT , Dutra BG , Boers AMM , et al . Association of reperfusion with brain edema in patients with acute ischemic stroke: a secondary analysis of the MR CLEAN trial. JAMA Neurol 2018;75:453–61. 10.1001/jamaneurol.2017.5162 29365017 PMC5885187

[5] Reinink H , Jüttler E , Hacke W , et al . Surgical decompression for space-occupying hemispheric infarction: a systematic review and individual patient meta-analysis of randomized clinical trials. JAMA Neurol 2021;78:208–16. 10.1001/jamaneurol.2020.3745 33044488 PMC7551237

[6] Kasner SE , Demchuk AM , Berrouschot Jörg , et al . Predictors of fatal brain edema in massive hemispheric ischemic stroke. Stroke 2001;32:2117–23. 10.1161/hs0901.095719 11546905

[7] Ong CJ , Gluckstein J , Laurido-Soto O , et al . Enhanced Detection of Edema in Malignant Anterior Circulation Stroke (EDEMA) score: a risk prediction tool. Stroke 2017;48:1969–72. 10.1161/STROKEAHA.117.016733 28487333 PMC5487281

[8] Jo K , Bajgur SS , Kim H , et al . A simple prediction score system for malignant brain edema progression in large hemispheric infarction. PLoS One 2017;12:0171425. 10.1371/journal.pone.0171425 PMC5298259 28178299

[9] Zi W , Wang H , Yang D , et al . Clinical effectiveness and safety outcomes of endovascular treatment for acute anterior circulation ischemic stroke in China. Cerebrovasc Dis 2017;44:248–58. 10.1159/000478667 28848210

[10] Tan JC , Dillon WP , Liu S , et al . Systematic comparison of perfusion-CT and CT-angiography in acute stroke patients. Ann Neurol 2007;61:533–43. 10.1002/ana.21130 17431875

[11] Broocks G , Kemmling A , Meyer L , et al . Computed tomography angiography collateral profile is directly linked to early edema progression rate in acute ischemic stroke. Stroke 2019;50:3424–30. 10.1161/STROKEAHA.119.027062 31665994

[12] Wu S , Yuan R , Wang Y , et al . Early prediction of malignant brain edema after ischemic stroke. Stroke 2018;49:2918–27. 10.1161/STROKEAHA.118.022001 30571414

[13] Kasner SE , Demchuk AM , Berrouschot J , et al . Predictors of fatal brain edema in massive hemispheric ischemic stroke. Stroke 2001;32:2117–23. 10.1161/hs0901.095719 11546905

[14] Huang X , Xu J , Yang K , et al . Blood pressure after endovascular thrombectomy and malignant cerebral edema in large vessel occlusion stroke. Front Neurol 2021;12:707275. 10.3389/fneur.2021.707275 34744962 PMC8564062

[15] Williams JL , Furlan AJ . Cerebral vascular physiology in hypertensive disease. Neurosurg Clin N Am 1992;3:509–20. 10.1016/S1042-3680(18)30642-9 1633475

[16] Hatashita S , Hoff JT , Ishii S . Focal brain edema associated with acute arterial hypertension. J Neurosurg 1986;64:643–9. 10.3171/jns.1986.64.4.0643 3950747

[17] Maïer B , Kubis N . Hypertension and its impact on stroke recovery: from a vascular to a parenchymal overview. Neural Plast 2019;2019:1–14. 10.1155/2019/6843895 PMC6815533 31737062

[18] Chamorro Ángel , Brown S , Amaro S , et al . Glucose modifies the effect of endovascular thrombectomy in patients with acute stroke. Stroke 2019;50:690–6. 10.1161/STROKEAHA.118.023769 30777000

[19] Lu G-D , Ren Z-Q , Zhang J-X , et al . Effects of diabetes mellitus and admission glucose in patients receiving mechanical thrombectomy: a systematic review and meta-analysis. Neurocrit Care 2018;29:426–34. 10.1007/s12028-018-0562-4 29946761

[20] Broocks G , Kemmling A , Aberle J , et al . Elevated blood glucose is associated with aggravated brain edema in acute stroke. J Neurol 2020;267:440–8. 10.1007/s00415-019-09601-9 31667625

[21] Thomalla G , Hartmann F , Juettler E , et al . Prediction of malignant middle cerebral artery infarction by magnetic resonance imaging within 6 hours of symptom onset: a prospective multicenter observational study. Ann Neurol 2010;68:435–45. 10.1002/ana.22125 20865766

[22] Li F , Silva MD , Liu KF , et al . Secondary decline in apparent diffusion coefficient and neurological outcomes after a short period of focal brain ischemia in rats. Ann Neurol 2000;48:236–44. 10.1002/1531-8249(200008)48:2&lt;236::AID-ANA14&gt;3.0.CO;2-7 10939575

[23] Kidwell CS , Saver JL , Starkman S , et al . Late secondary ischemic injury in patients receiving intraarterial thrombolysis. Ann Neurol 2002;52:698–703. 10.1002/ana.10380 12447922

[24] Broocks G , Hanning U , Flottmann F , et al . Clinical benefit of thrombectomy in stroke patients with low aspects is mediated by oedema reduction. Brain 2019;142:1399–407. 10.1093/brain/awz057 30859191

[25] Zi W , Qiu Z , Li F , et al . Effect of endovascular treatment alone vs intravenous alteplase plus endovascular treatment on functional independence in patients with acute ischemic stroke: the DEVT randomized clinical trial. JAMA 2021;325:234–43. 10.1001/jama.2020.23523 33464335 PMC7816099

[26] Yang P , Zhang Y , Zhang L , et al . Endovascular thrombectomy with or without intravenous alteplase in acute stroke. N Engl J Med 2020;382:1981–93. 10.1056/NEJMoa2001123 32374959

[27] Smith EE , Zerna C , Solomon N , et al . Outcomes after endovascular thrombectomy with or without alteplase in routine clinical practice. JAMA Neurol 2022;79:e221413. 10.1001/jamaneurol.2022.1413 PMC9194745 35696198

[28] Bae S , Ahn SS , Kim BM , et al . Hyperattenuating lesions after mechanical thrombectomy in acute ischaemic stroke: factors predicting symptomatic haemorrhage and clinical outcomes. Clin Radiol 2021;76:80.e15-80.e23. 10.1016/j.crad.2020.08.021 32950255

[29] Nikoubashman O , Reich A , Gindullis M , et al . Clinical significance of post-interventional cerebral hyperdensities after endovascular mechanical thrombectomy in acute ischaemic stroke. Neuroradiology 2014;56:41–50. 10.1007/s00234-013-1303-1 24306553

