# Supplementary material for: The ACORNS grading scale: a novel tool for the prediction of malignant brain edema after endovascular thrombectomy
Source: J Neurointerv Surg. 2022 Oct 7;15(e2):e190–7. doi: 10.1136/jnis-2022-019404 (PMC10646923; doi:10.1136/jnis-2022-019404)
Supplement: Supplementary data [file jnis-2022-019404supp001.pdf]

Supplementary Materials

The ACORNS grading scale: A novel tool for the prediction of malignant brain edema after endovascular thrombectomy

RESULTS

Post-hoc sensitivity analysis

Considering that hemorrhagic transformation may affect the assessment of MBE, we excluded the symptomatic intracranial hemorrhage (sICH) in the derivation cohort (97/914, 10.6%). Then, we were randomly assigned to either the training set (n=571) or the internal validation set (n=246). In the training cohort, we did not find the difference of the rate of MBE between the patients who received intravenous alteplase plus EVT and the patients who received EVT alone.

The logistic regression analysis showed that baseline ASPECT scores, collateral circulation, reperfusion status, and occlusion site were independent predictors for the occurrence of MBE (Table 1). The p value of the Hosmer–Lemeshow test was 0.599. The AUC was 0.843 (95% CI 0.801-0.884, p <0.01).

Huang X, *et al. J NeuroIntervent Surg* 2023; 15:e190–e190. doi: 10.1136/jnis-2022-019404

Table I Missing Data of The Cohorts.

	Training Cohort n=643	Internal Validation Cohort n=271	External Validation Cohort n=502
Age, y, mean (SD)			
Men, n (%)			
Hypertension, n (%)			
Diabetes, n (%)			
Atrial fibrillation, n (%)			1
Baseline SBP, mmHg, media (IQR)	3	4	
Baseline DBP, mmHg, media (IQR)	3	4	
Baseline NIHSS score, media (IQR)			
Baseline ASPECT score, media (IQR)	3		
IVT, n (%)			
TOAST classification, n (%)			
Atherosclerotic			
Cardioembolic			
Others			
OTP, min, media (IQR)			
OTR, min, media (IQR)	3		3
Collateral status, n (%)			
Poor			
Good			
First treatment, n (%)	1	2	
Rescue therapy, n (%)	1	2	
Tandem, n (%)			
Occlusion site, n (%)			
mTICI 2b-3, n (%)			
MBE, n (%)			
FBG, mmol/L, mean (SD)	27	23	
LDL, mmol/L, mean (SD)	58	40	90
BUN, mmol/L, mean (SD)	25	17	46
Cr, µmol/L, mean (SD)	25	17	46

ASPECT indicates Alberta Stroke Program Early CT; BUN, blood urea nitrogen; Cr, creatinine; DBP, diastolic blood pressure; FBG, fast blood glucose; ICA, internal carotid artery; IVT, intravenous thrombolysis; IQR, interquartile range; LDL, low-density lipoprotein; mTICI, modified Thrombolysis in Cerebral Infarction; MCA, middle cerebral artery; MBE, malignant brain edema; NIHSS, National Institutes of Health Stroke Scale; OTP, symptom onset to groin puncture time; OTR, time from stroke onset to recanalization; SBP, systolic blood pressure; SD, standard deviation; TOAST, the Trial of ORG 10172 in Acute Stroke Treatment.

Table II Results of the univariable and multivariable logistic regression for MBE after EVT in the training cohort excluded sICH.

	Univariable			Multivariable		
	MBE n=95	Non-MBE n=476	P Value	Regression Coefficient	OR (95% CIs)	P Value
Age, y, mean (SD)	67.8 (11.1)	66.0 (11.4)	0.137			
Men, n (%)	56 (58.9)	303 (63.7)	0.386			
Hypertension, n (%)	72 (75.8)	311 (65.3)	**0.048**	0.478	1.612 (0.892-2.914)	0.114
Diabetes, n (%)	16 (16.8)	87 (18.3)	0.740			
Atrial fibrillation, n (%)	50 (52.6)	208 (43.7)	0.110			
Baseline SBP, mmHg, media (IQR)	150 (130, 162)	149 (129, 160)	0.535			
Baseline DBP, mmHg, media (IQR)	82 (70,91)	81 (74, 91)	0.659			
Baseline NIHSS score, media (IQR)	17 (15, 20)	15 (12, 19)	**< 0.001**	0.036	1.037 (0.989-1.087)	0.131
Baseline ASPECT score, media (IQR)	8 (5, 8)	9 (8, 10)	**< 0.001**	-0.292	0.747 (0.66-0.837)	**< 0.001**
IVT, n (%)	26 (27.4)	99 (20.8)	0.157			
TOAST classification, n (%)			0.154			
Atherosclerotic	181 (38)	28 (29.5)				
Cardioembolic	234 (49.2)	57 (60)				
Others	61 (12.8)	10 (10.5)				
OTP, min, media (IQR)	267 (210, 336)	273 (216, 350)	0.381			
OTR, min, media (IQR)	370 (292, 453)	351 (287, 445)	0.389			
Collateral status, n (%)			**< 0.001**	-1.234	0.291 (0.156-0.543)	**< 0.001**
Poor	79 (83.2)	225 (47.3)				
Good	16 (16.8)	251 (52.7)				
First-line treatment, n (%)			0.234			
Stent retriever	63 (67)	312 (65.8)				
Contact aspiration	23 (24.5)	94 (19.8)				
Angioplasty or stent	8 (8.5)	68 (14.3)				
mTICI 2b-3, n (%)	61 (64.2)	410 (86.1)	**< 0.001**	-0.879	0.415 (0.231-0.746)	**0.003**
Tandem, n (%)	13 (13.7)	56 (11.8)	0.600			
Occlusion site, n (%)			**< 0.001**			
MCA	28 (29.5)	292 (61.3)		-1.115	0.328 (0.190-0.565)	**< 0.001**

Huang X, *et al. J NeuroIntervent Surg* 2023; 15:e190–e190. doi: 10.1136/jnis-2022-019404

ICA	67 (70.5)	184 (38.7)				
FBG, mmol/L, mean (SD)	8.22 (3.2)	6.98 (5.44)	**< 0.001**	0.031	1.031 (0.994-1.070)	0.097
LDL, mmol/L, mean (SD)	2.37 (0.89)	2.38 (0.75)	0.647			
BUN, mmol/L, mean (SD)	6.95 (4.05)	5.86 (2.30)	**0.017**	0.078	1.081 (0.993-1.176)	0.072
Cr, µmol/l, mean (SD)	86.19 (56.04)	80.58 (32.75)	0.389			

ASPECT, Alberta Stroke Program Early CT; BUN, blood urea nitrogen; Cr, creatinine; CIs, confidence intervals; DBP, diastolic blood pressure; FBG, fasting blood glucose; ICA, internal carotid artery; IVT, intravenous thrombolysis; IQR, interquartile range; LDL, low-density lipoprotein; mTICI, modified thrombolysis in cerebral infarction; MCA, middle cerebral artery; MBE, malignant brain edema; NIHSS, National Institutes of Health Stroke Scale; OTP, symptom onset to groin puncture time; OTR, time from stroke onset to recanalization; OR, odds ratios; SBP, systolic blood pressure; SD, standard deviation; sICH, symptomatic intracranial hemorrhage; TOAST, the Trial of ORG 10172 in Acute Stroke.

Huang X, *et al. J NeuroIntervent Surg* 2023; 15:e190–e190. doi: 10.1136/jnis-2022-019404

Table III The MBE rates according to ACORNS scores in three cohorts.

ACORNS scores	Training Cohort		Internal Validation Cohort		External Validation Cohort	
	MBE rate	MBE rate (95%CI), %	MBE rate	MBE rate (95%CI), %	MBE rate	MBE rate (95%CI), %
0	0/6	-	0/10	-	0/7	-
1	0/52	-	0/19	-	1/34	2.9 (0.2-17.1)
2	2/67	3 (0.5-11.3)	1/23	4.4 (0.2-23.9)	3/53	5.7 (1.5-16.6)
3	2/83	2.4 (0.4-9.2)	1/23	4.4 (0.2-23.9)	4/52	7.7 (2.5-19.4)
4	6/91	6.6 (2.7-14.3)	3/41	7.3 (1.9-21)	7/59	11.9 (5.3-23.5)
5	12/85	14.1 (7.8-23.8)	1/28	3.6 (0.1-20.2)	15/57	26.3 (15.9-39.9)
6	17/64	26.6 (16.7-39.3)	8/31	25.8 (12.5-44.9)	13/49	26.5 (15.4-41.3)
7	22/65	33.9 (22.9-46.8)	8/32	25 (12.1-43.8)	18/61	29.5 (18.9-42.7)
8	24/58	41.4 (28.9-55)	4/21	19.1 (6.3-42.6)	22/51	43.1 (29.6-57.7)
9	23/36	63.9 (46.2-78.7)	11/17	64.7 (38.6-84.7)	18/34	52.9 (35.4-69.8)
10	13/20	65 (40.9-83.7)	10/14	65 (40.9-84.7)	8/18	44.4 (22.4-68.7)
11-12	10/11	90.1 (57.1-99.5)	9/11	81.8 (47.8-96.8)	13/19	68.4 (43.5-86.4)
13-15	4/5	80 (29.9-98.9	1/1	100 (54.6-100)	8/8	100 (59.8-100)

Huang X, *et al. J NeuroIntervent Surg* 2023; 15:e190–e190. doi: 10.1136/jnis-2022-019404

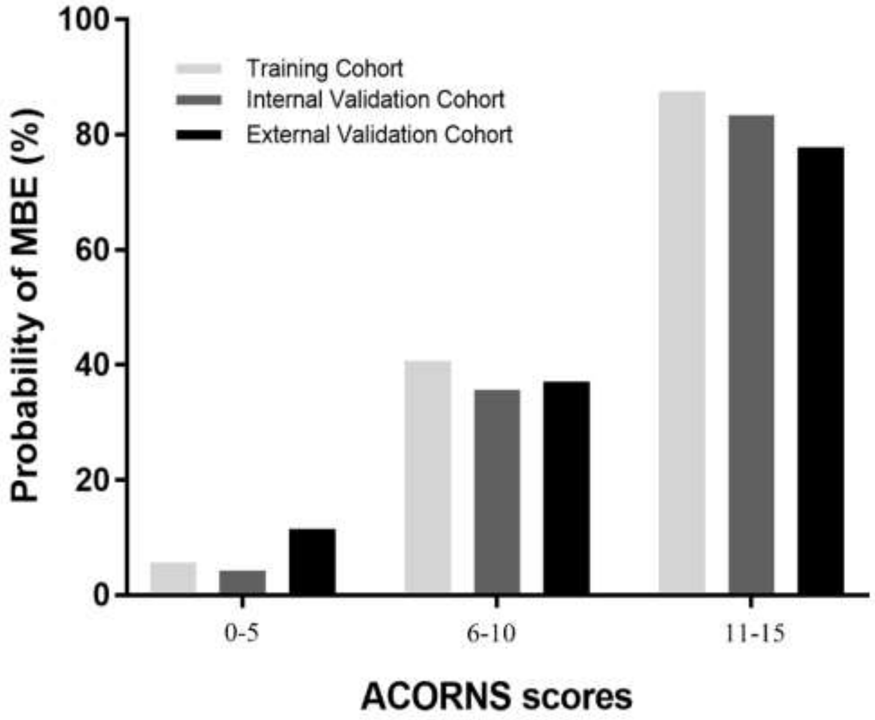

Figure I. Probability of MBE according to ACORNS scores. Patients were grouped according to their ACORNS scores (0–5, 6–10 and 11–15). The MBE rate increased with the ACORNS scores as in these groups.
